# Correction: Self-reported musculoskeletal disorders questionnaire for agriculturists: An online self-assessment tool development

**DOI:** 10.1371/journal.pone.0285304

**Published:** 2023-04-27

**Authors:** Worawan Poochada, Sunisa Chaiklieng

The second author’s name is spelled incorrectly. The correct name is: Sunisa Chaiklieng. The correct citation is: Poochada W, Chaiklieng S (2022) Self-reported musculoskeletal disorders questionnaire for agriculturists: An online self-assessment tool development. PLoS ONE 17(12): e0277548. https://doi.org/10.1371/journal.pone.0277548
