# Supplementary figures and images for: Self-reported musculoskeletal disorders questionnaire for agriculturists: An online self-assessment tool development
Source: PLoS One. 2022 Dec 21;17(12):e0277548. doi: 10.1371/journal.pone.0277548 (PMC9770398; doi:10.1371/journal.pone.0277548)

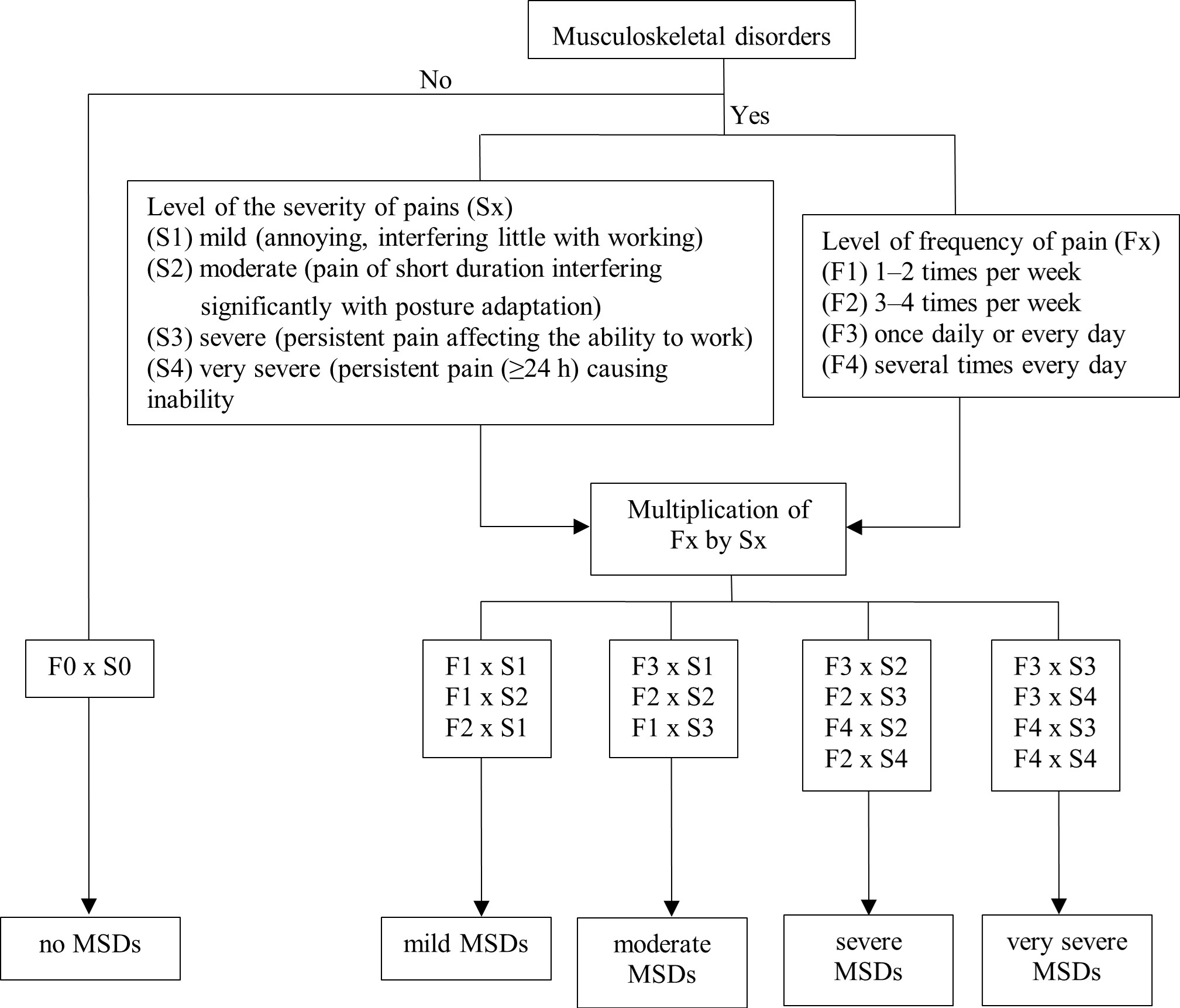

Supplement: S1 Fig — (JPG) [file pone.0277548.s001.jpg]

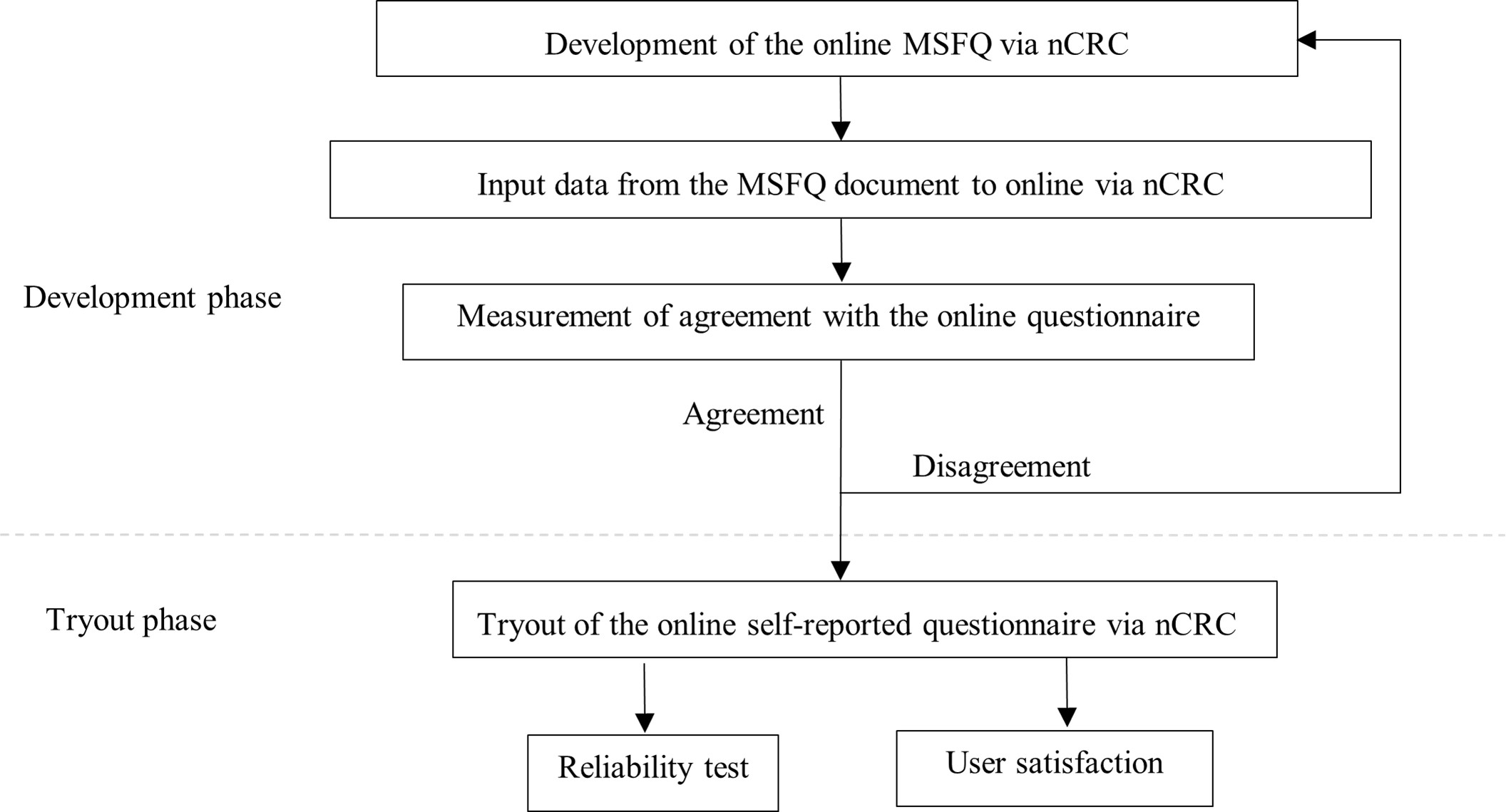

Supplement: S2 Fig — (JPG) [file pone.0277548.s002.jpg]
