# Supplementary material for: Self-reported musculoskeletal disorders questionnaire for agriculturists: An online self-assessment tool development
Source: PLoS One. 2022 Dec 21;17(12):e0277548. doi: 10.1371/journal.pone.0277548 (PMC9770398; doi:10.1371/journal.pone.0277548)
Supplement: S1 Table — (DOCX) [file pone.0277548.s003.docx]

**Table 1. Number (%) of users based on level of satisfaction with the online MSFQ**

| **Topic of satisfaction** | **Level of satisfaction with the self-reported MSDs questionnaire** | | | | | |
| --- | --- | --- | --- | --- | --- | --- |
|  | **VS** | **S** | **N** | **US** | **VUS** | **Mean ± S.D.** |
| **1. Information** | | | | | | |
| 1.1. Easily understood language for usage | 22 (68.8) | 8 (25.0) | 2 (6.3) | 0 (0.00) | 0 (0.00) | 4.63 ± 0.61 |
| 1.2. The questions are continuous | 23 (71.9) | 9 (28.1) | 0 (0.00) | 0 (0.00) | 0 (0.00) | 4.72 ± 0.46 |
| Average | | | | | | 4.67 ± 0.54 |
| **2. Program design** | | | | | | |
| 2.1. Easy access to appraisals via mobile | 20 (62.5) | 10 (31.3) | 2 (6.3) | 0 (0.00) | 0 (0.00) | 4.56 ± 0.62 |
| 2.2. The font size is suitable | 21 (65.6) | 8 (25.0) | 3 (9.4) | 0 (0.00) | 0 (0.00) | 4.56 ± 0.67 |
| 2.3. Easy to use | 21 (65.6) | 9 (28.1) | 2 (6.3) | 0 (0.00) | 0 (0.00) | 4.59 ± 0.61 |
| 2.4. It has a sequence of steps | 23 (71.9) | 9 (28.1) | 0 (0.00) | 0 (0.00) | 0 (0.00) | 4.72 ± 0.46 |
| Average | | | | | | 4.61 ± 0.59 |
| **3. Benefits** | | | | | | |
| 3.1. Able to self-report MSDs | 24 (75.0) | 8 (25.0) | 0 (0.00) | 0 (0.00) | 0 (0.00) | - 1. ± 0.44 |
| 3.2. Able to know the MSDs results immediately | 25 (78.1) | 7 (21.9) | 0 (0.00) | 0 (0.00) | 0 (0.00) | - 1. ± 0.42 |
| 3.3. Stretching muscles was advised | 26 (81.3) | 6 (18.8) | 0 (0.00) | 0 (0.00) | 0 (0.00) | 4.81 ± 0.40 |
| Average | | | | | | 4.73 ± 0.46 |
| **4. Overall** | 20 (62.5) | 11 (34.4) | 1 (3.1) | 0 (0.00) | 0 (0.00) | 4.59 ± 0.56 |

VS = Very satisfied, S = Satisfied, N = Neutral, US = Unsatisfied, VUS = Very unsatisfied
