# Supplementary material for: Self-reported musculoskeletal disorders questionnaire for agriculturists: An online self-assessment tool development
Source: PLoS One. 2022 Dec 21;17(12):e0277548. doi: 10.1371/journal.pone.0277548 (PMC9770398; doi:10.1371/journal.pone.0277548)
Supplement: S1 Appendix. An online MSFQ — (PDF) [file pone.0277548.s004.pdf]

Appendix A: An online MSFQ

(applied from Chaiklieng S. Health risk assessment on musculoskeletal disorders among potato-chip processing workers. PLoS One 2019, 14, e0224980. https://doi.org/10.1371/journal.pone.0224980)

Step 1: Users clicked on the icon to access the MSFQ (1). The first page showed a body diagram so that users could consider which areas of pain, aching or discomfort had been caused by working or occurred during work in the past month (2). If the user had no pain, aching or discomfort, the MSDs level was immediately reported as "no MSDs" and shown on the screen (6).

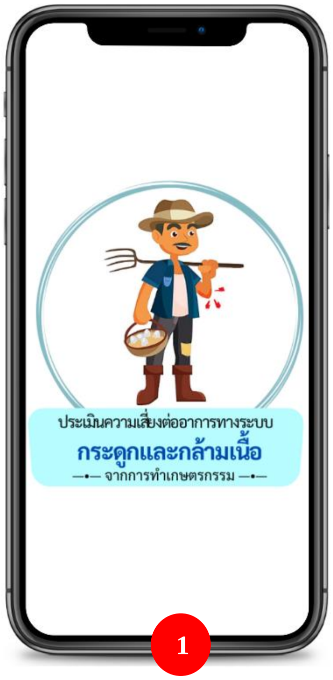

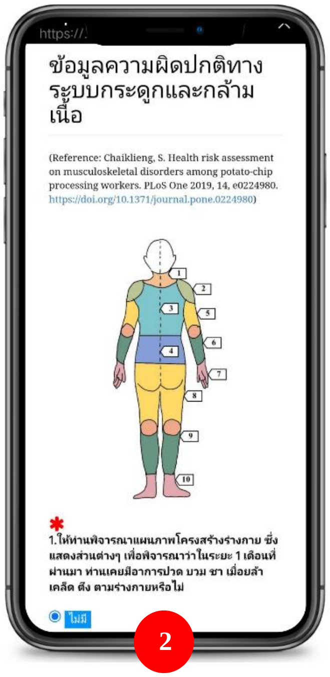

Step 2: Following the display of the body diagram (2), ten bodily parts were listed in the form of number and text so the user could indicate which ones they might be experiencing pain, aching, or discomfort in (3). If users felt aches, pains, or discomfort in particular body regions, the level of severity and frequency of such pain was requested (4).

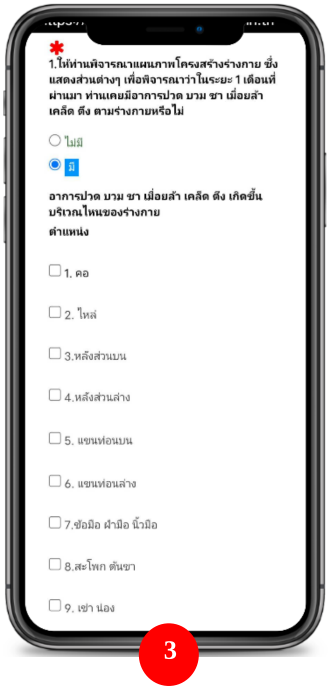

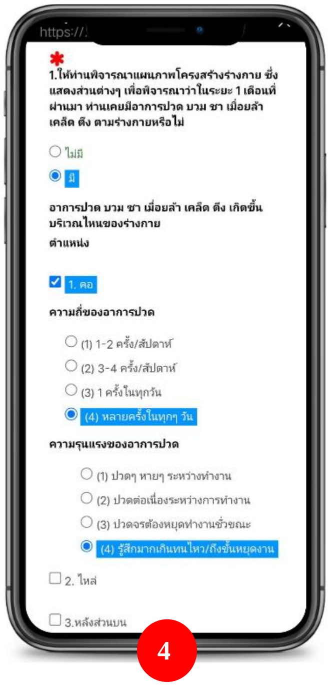

Step 3: Questions (5) regarding "work-related pain in the last 7 days" and "confirmation of the work-related pain in the past month" were asked. Then, answers to the questions were sent, the MSDs level was immediately reported (6) and the MSDs level and recommendations for prevention and control were shown.

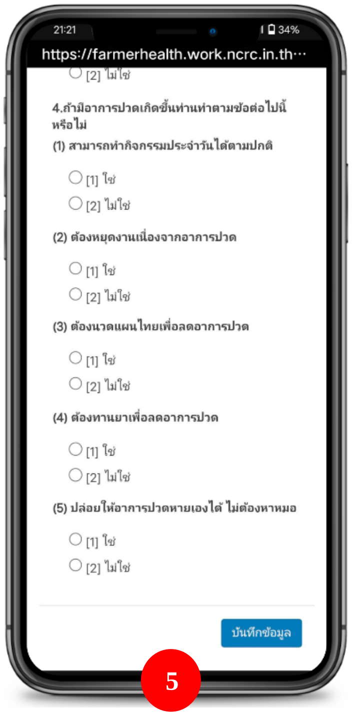

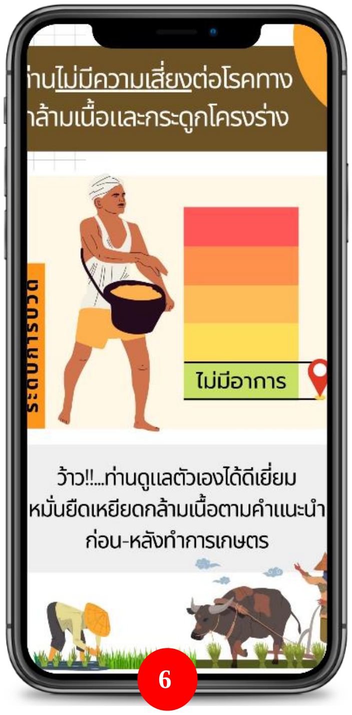

Note1: The MSDs levels according to the MSFQ were classified into five levels which were immediately reported, as shown below.

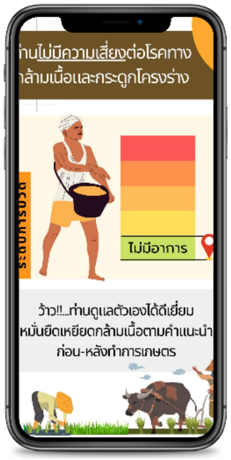

no MSDs

(score: 0 points)

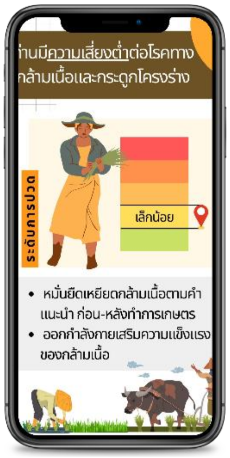

mild MSDs

(score: 1-2 points)

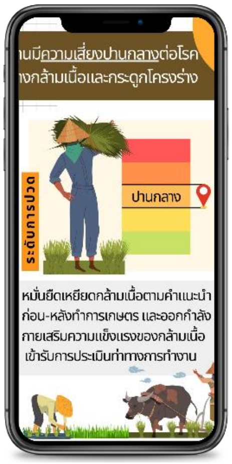

moderate MSDs

(score: 3-4 points)

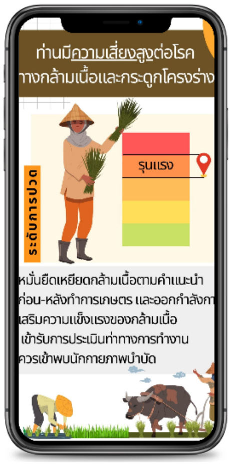

severe MSDs

(score: 5-8 points)

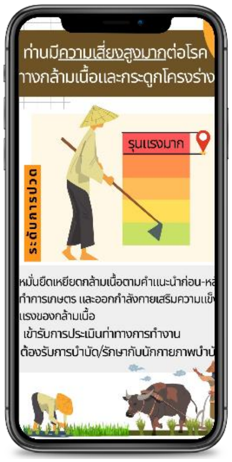

very severe MSDs

(score: 9-16 points)
