# Supplementary material for: Self-reported musculoskeletal disorders questionnaire for agriculturists: An online self-assessment tool development
Source: PLoS One. 2022 Dec 21;17(12):e0277548. doi: 10.1371/journal.pone.0277548 (PMC9770398; doi:10.1371/journal.pone.0277548)
Supplement: Attachment — Submitted filename: 2. Responsed to reviewer sc- 09102022.docx [file pone.0277548.s005.docx]

**Response to Reviewer 1**

**1. Overall**

**1.1)** The manuscript is very good and valuable, and the authors have done a lot of effort, but for me, the question is whether self-report and questionnaire without clinical examination accurately determine that the disease is caused by work?

**Author to respond reviewer:** Thank you very much for your kind understanding that this work was done a lot of effort, and we wish to contribute this valuable product to agricultural workers and related occupation around the world to be useful as soon as we can.

It was still limitation of self-report assessment on work-related MSDs. We identified the limitation at discussion section. However, the 4^th^ question of MSFQ was confirmation of the work-related pain in the past month, from the previous study of the MSDs Severity and Frequency Questionnaire (MSFQ) (Chaiklieng 2019, PlosONE….[9] and was used by the previous screening of MSDs (available at  <https://www.mdpi.com/article/10.3390/safety8030061/s1>, [14]. The previous studies used also this tool for self-report that could determine the occupational MSDs, and this study provide the available online tool that could be accessed easier and report result in such a real time.

**1.2)** the following are suggested for improvement:

**(1)** There are some grammatical issues throughout the paper that would benefit from an additional review and in other words grammar needs to be corrected throughout the manuscript.

**Author to respond reviewer:** We are already proofreading before resubmission by native speaker.

**(2)** Introduction needs to be rewritten.

**Author to respond reviewer:** we revised with yellow highlight.

**(3)** The Materials and methods is long and vague, it is better to be concise and clear.

**Author to respond reviewer:** we revised with yellow highlight.

**2. Abstract**

**2.1)** The research method (Statistical population, Sampling method, etc.) should be written.

**Author to respond reviewer:** We already add with yellow highlight. However, abstract not exceed 300 words.

**2.2)** What tests have been used?

**Author to respond reviewer:** We already add with yellow highlight. However, abstract not exceed 300 words.

**3. Introduction**

**3.1)** The introduction and problem statement is very brief and requires further explanation.

**Author to respond reviewer:** we revised with yellow highlight in the first paragraph to be more explanation.

**3.2)** The importance of the topic should be explained in such a way that the designed tool can determine whether the disease is caused by the work or not.

**Author to respond reviewer:** we revised with yellow highlight which explain the MSFQ could determine the disease caused by work from the previous study and the screening phase in the second paragraph.

**3.3)** It would be better to include a literature review.

**Author to respond reviewer:** we revised with yellow highlight in the second paragraph to include the literature review.

**3.4)** Research innovation to be written

**Author to respond reviewer:** we revised with yellow highlight in the last paragraph of research innovation..

**4. Materials and methods**

**4.1)** The research method should be expressed in a more coherent and clear way.

**Author to respond reviewer:** we revised with yellow highlight for more coherent method and clear way.

**5. Discussions**

**5.1)** The discussion should be written to be reflect the results

**Author to respond reviewer:** we revised with yellow highlight in the part of discussion to be reflect the results.

**6. Conclusions and suggestions**

**6.1)** The conclusion is very brief so it should be reinforced

**Author to respond reviewer:** we revised with yellow highlight more important points from the study

**6.2)** Research limitations, the strengths and weaknesses of the study, suggestions should be written

**Author to respond reviewer:** we revised with yellow highlight in the second paragraph for the limitation and the strength of the study.

**7. Conclusions and suggestions**

**7.1)** The reference should be given at the end of the manuscript according to the journal format.

**Author to respond reviewer:** we followed by manuscript body formatting guidelines, modified April 2021.

**7.2)** Match the references inside the text and at the end of the text.

**Author to respond reviewer:** we rechecked all reference and format.

**The Review Report (Reviewer 2)**

**Comments and Suggestions for Authors**

The tool will help in self reporting of MSDs in agriculture and will help to cover workers all around the globe. It is always not possible to find musculoskeletal problems of farm workers experimentally.

**Author to respond reviewer:** Thank you for your kind comments and suggestions, this work was very hard working and needed a lot of effort and budget, and we hope that the fast publication will be very useful to move on the next step for contribution work to other agriculturist around around the globe
